# Oligodendrocytes in a Nutshell

**DOI:** 10.3389/fncel.2015.00340

**Published:** 2015-09-01

**Authors:** John-Paul Michalski, Rashmi Kothary

**Affiliations:** ^1^Ottawa Hospital Research Institute, Ottawa, ON, Canada; ^2^Department of Cellular and Molecular Medicine, University of Ottawa, Ottawa, ON, Canada; ^3^Department of Medicine, University of Ottawa, Ottawa, ON, Canada; ^4^Centre for Neuromuscular Disease, University of Ottawa, Ottawa, ON, Canada

**Keywords:** myelin, myelination, OPC, glial cell, cytoskeleton, actin, microtubule

## Abstract

Oligodendrocytes are the myelinating cells of the central nervous system (CNS). While the phrase is oft repeated and holds true, the last few years have borne witness to radical change in our understanding of this unique cell type. Once considered static glue, oligodendrocytes are now seen as plastic and adaptive, capable of reacting to a changing CNS. This review is intended as a primer and guide, exploring how the past 5 years have fundamentally altered our appreciation of oligodendrocyte development and CNS myelination.

## Introduction

In the central nervous system (CNS), oligodendrocytes (OLs) are the glial-subtype responsible for myelin production. The OL’s diminutive size belies the scale of myelin membrane produced – membrane capable of ensheathing dozens of axons in multiple layers, all the while contracting and expelling cytoplasm to generate mature myelin. Today, we take this knowledge for granted. It is easy to forget that until the mid-1950s, many researchers believed the myelin sheath to be an axonal creation, and not the natural product of glia (Geren and Raskind, 1953; Geren, 1954). This notion that glia played second fiddle unfortunately persisted. Often viewed as little more than “glue” holding the nervous system together, OLs have had difficulty in ridding themselves of this namesake and driving interest in the scientific community. Researchers have only recently begun to truly dissect and understand this unique cell type. Advances in imaging technology and model systems have allowed scientists unprecedented access to the inner workings of the OL. From roles in axonal maintenance, survival, and adaptation (Nave, 2010b; Fünfschilling et al., 2012; Lee et al., 2012b; Gibson et al., 2014; Tomassy et al., 2014) to sculpting higher order neuronal circuitry (McKenzie et al., 2014; Tomassy et al., 2014), our understanding and appreciation of the OL have never been greater.

This review aims to introduce the reader to fundamental aspects of OL development and myelination – taking the OL from simple bipolar progenitor to fully mature myelin-producing cell.

## Oligodendrocytes in CNS Myelination

From start to finish, the life of an OL is defined by four distinct phases: (1) the birth, migration, and proliferation of oligodendrocyte precursor cells (OPCs), a process occurring in waves, followed by (2) morphological differentiation – the OL establishes an expansive network of processes, (3) axonal contact, leading to ensheathment and generation of compact myelin around target axons, and (4) long-term trophic and metabolic support of the encased axon.

### Oligodendrocyte precursor cells: Ng2-positive cells and polydendrocytes

Oligodendrocyte precursor cells (OPCs) are primarily identified by the expression of two key markers: the chondroitin sulfate proteoglycan NG2 and the platelet-derived growth factor receptor alpha (PDGFRα). In recent years, there has been some confusion with regards to the identity and nomenclature assigned to NG2-expressing cells. They have been called both OPCs and/or “polydendrocytes,” and are considered by many to be the fourth major glial cell of the CNS (Nishiyama et al., 2014). The confusion is due in large part to the cell’s multipotency. While predominantly a pool for OLs – and hence considered OPCs – NG2-expressing cells also generate protoplasmic astrocytes and a small number of region-specific neurons (though the latter is hotly debated) (Rivers et al., 2008; Zhu et al., 2008; Guo et al., 2010; Kang et al., 2010). Recent evidence further suggests that NG2 cells are capable of receiving and reacting to neuronal input, though no definitive biological function has been ascribed [reviewed by Hill and Nishiyama (2014)]. To generate consensus and acknowledge the NG2 population as distinct, an umbrella term, polydendrocyte, was adopted. The term polydendrocyte encompasses all NG2-expressing cells in the CNS, including those whose function is not necessarily linked to OLs (Nishiyama et al., 2014). For the purpose of this review, we will refer to NG2 cells as OPCs, as our primary concern lies with the myelination process.

### OPCs in development: From embryo to early post-natal period

Central nervous system birthing of the OL lineage occurs in distinct waves through time and space. In the mouse spinal cord, OLs begin life as migrating precursors originating in the ventral ventricular zone at E12.5 (Warf et al., 1991; Lu et al., 2002; Richardson et al., 2006). A second wave, though much smaller, follows from the dorsal ventricular zone approximately 2 days later, with a third, smaller still, originating postnatally (Cai et al., 2005; Fogarty et al., 2005; Vallstedt et al., 2005; Sevc et al., 2014). Similarly, in the mouse brain, three separate OPC waves (also beginning at E12.5) eventually define the mature OL population. These temporal migrations compete for space (Kessaris et al., 2006). Importantly, OPCs display compensatory redundancy – when any single wave is destroyed, OPCs present before or after fill the void and development proceeds unaffected (Kessaris et al., 2006). Interestingly, the idea of “oligodendrocytes at war,” either as competition between populations or individual cells, appears thematic of OL biology (Richardson et al., 2006). For example, the CNS produces an overabundance of OPCs across all regions during development. A large percentage die during the myelination process, as the cells compete for limited axonal/astrocytic survival factors (Barres et al., 1992; Trapp et al., 1997; Barres and Raff, 1999). In zebrafish, OPC laser ablation results in local expansion of neighboring progenitors. Through process extension, OPCs first sample then settle the vacated space, thereby maintaining a progenitor landscape primed for myelination (Kirby et al., 2006).

A recent study takes modulation one step further, focusing on environmental factors affecting OPC outcome during a critical temporal window – the period between final cell division and terminal OL differentiation (Hill et al., 2014). For example, following CNS injury, the lag time between progenitor division and OL differentiation/myelin production can be shortened, highlighting the OPC’s ability to accelerate through the differentiation gamut. In contrast, the CNS can trigger OPC cell death when there is a reduced need for myelination. Case in point, following whisker clipping (a form of sensory deprivation), young mice require fewer OLs in the now inactive associated somatosensory cortex. As a consequence, a greater number of OPCs in the region, caught between division and differentiation, undergo apoptosis (Hill et al., 2014). Clearly, mechanisms have evolved that allow for effective myelination capacity even under duress, or, in the case of sensory deprivation, adapt OL production to match neuronal activity. As we will see, this notion can be expanded to include mature myelin-producing OLs as they compete for bare axons.

### OPCs in the adult

A substantial number of OPCs persist in the adult brain. Here, they comprise the largest population of dividing cells, each subsisting within its own unique non-overlapping domain (Dawson et al., 2003; Hughes et al., 2013). Again, in the vein of competition and balance, recent work from the Bergles laboratory beautifully captures the adult OPC population in a state of controlled equilibrium (Hughes et al., 2013). With the aid of reporter mice and cranial windows, live recorded OPCs were shown to exist within unique spatial pockets, established through inter-repulsive cues with other progenitors. OPCs were highly exploratory, sampling their environment in search of unoccupied space. OPC loss led to rapid invasion/division by neighboring progenitors, ensuring a spatially crafted balance in the aged brain similar to development. It paints the picture of a web-like network, with unique micro-domains strung throughout. Pulling on any “string” triggers local, or in the case of injury, global reverberations, thereby forcing a response – be it proliferation, migration, or repair.

While functional nuances abound (and remain highly debated, see previous section on NG2 cells), progenitor cells are generally thought responsible for myelin maintenance, both in normal and diseased brains (Rivers et al., 2008; Kang et al., 2010; Young et al., 2013). In the non-diseased brain, OPCs are actively recruited for myelin remodeling, and possibly, *de novo* myelination (Young et al., 2013). Here, they replace naturally dying OLs, generating myelin for white matter tract maintenance following turnover, or, in the case of *de novo*, are thought to be molded in response to adaptive neural plasticity. In support of *de novo* adaptation, stimulated and therefore electrically active neurons have recently been shown capable of driving OPC proliferation and oligodendrogenesis *in vivo* (Gibson et al., 2014). The change in myelin capacity within the activated circuit, in this case the premotor cortex, led to motor improvement (Gibson et al., 2014).

### Oligodendrocytes: From immature to mature myelin-producing cell

**Figure 1:**
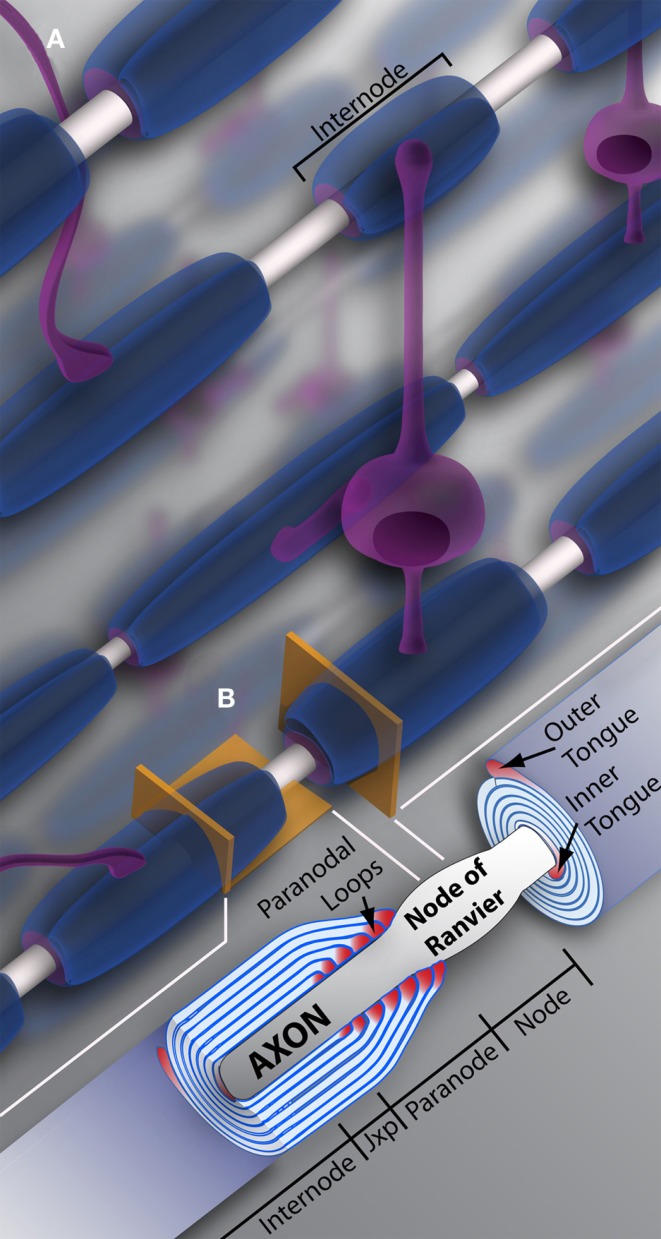
**Oligodendrocytes and myelin**. **(A)** Schematic representation of oligodendrocytes and myelin internodes in the CNS. As depicted, a typical oligodendrocyte (in purple) is capable of myelinating multiple axons (depicted in light gray). The oligodendrocyte will extend numerous processes, each ending in a myelin internode, depicted here in blue. Internodes within the same region, even along the same axon, can differ in length and/or size, providing the neuronal network or individual neurons with unique myelin profiles. **(B)** Vertical and longitudinal sections through a myelin sheath. Note the cytoplasm rich inner and outer tongues. The axon is unmyelinated and bulges at the Node of Ranvier, also referred to as the node. Swollen cytoplasmic rich paranodal loops (cytoplasm here denoted by the color pink) abut the axon, delineating the paranode. Paranodal loops anchor the myelin membrane to the axolemma through axo-glial junctions. Junctions are composed of axolemma and OL-based proteins, notably contactin, contactin-associated protein (Caspr), and neurofascin 155. These proteins hook together, keeping the loops and axolemma in close proximity. The paranode gives way to the juxtaparanode (juxtaparanode, as it is in juxtaposition with the paranode), a small area rich in K^+^ channels. Next is the internode, that can refer both to the myelin sheath as a whole **(A)**, or the space under compact myelin between juxtaparanodes. Jxp = juxtaparanode.

**Figure 2:**
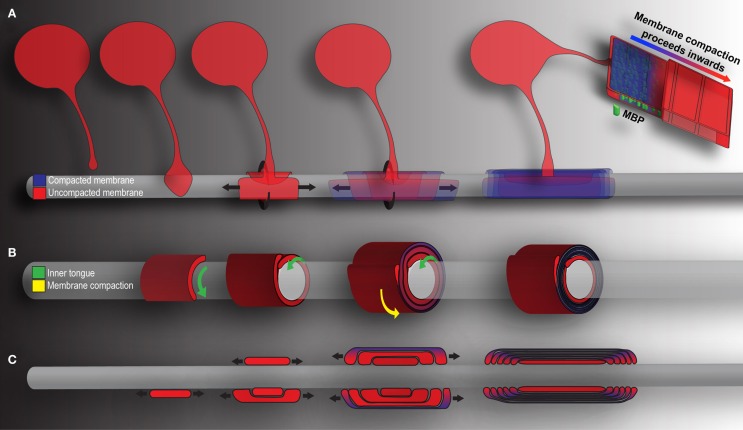
**Mechanics of CNS myelination**. Presented is a simplified schematic of OL-mediated myelination from various points of view. Cytoplasm rich, newly deposited membrane is represented in red, while compact membrane is shown in blue. **(A)** OLs extend a “growth-cone-like” tip seeking bare axons. Upon axonal contact, the tip expands, forming a triangular shaped membrane. Membrane growth occurs through simultaneous radial (around) and lateral (along) expansion. The growing inner tongue pushes radially under pre-existing membrane, depositing new membrane in its wake. During this process, the outermost layers of membrane (those first deposited) fill with MBP and compaction begins. Compaction and associated MBP clustering follows behind the growing inner tongue, zippering the newly formed membrane shut. When all necessary membrane has been deposited, the inner tongue halts growth and enters a period of stasis (shrinking as a result). It can be reactivated at a later time point to deposit additional myelin as required by the axon. **(B)** A cross-section through myelin membrane as presented in **(A)**. Again, the inner tongue grows radially underneath pre-existing membrane. Membrane compaction occurs first in the older, outermost membrane layers and proceeds inwards. **(C)** Longitudinal representation of myelin membrane as presented in **(A)**. Note that the lateral growth and eventual formation of paranodal loops as the outermost layers compact to form mature myelin.

Oligodendrocyte precursor cell differentiation is characterized by a rapid increase in morphological complexity (branching of the cell) followed by expansion of uncompacted myelin membrane. The nascent OL is required to produce an astronomic volume of membrane – upwards of 50 myelin sheaths in some CNS regions (see Figure 1) (Chong et al., 2012). The gamut of differentiation, presented in a simplified manner, occurs as follows: in early stages, the OL extends multiple highly ramified processes that contact nude axons and trigger myelination. Process tips are dynamic, and while once considered simple filopodia, there is now recognition within the field for “growth-cone-like” structures directing, guiding, and retracting the developing branched network (Fox et al., 2006; Sloane and Vartanian, 2007; Czopka et al., 2013). The OL process expands upon axonal contact, wrapping the axon in concentric layers of membrane (see Figure 2). The membrane compacts, expelling cytoplasm, and mature myelin is born (Snaidero et al., 2014). Individual myelin segments, still connected to the OL soma, are termed internodes (see Figure 1A). Unlike most schematics depicting sheaths as uniform and ordered, internodes originating from a single OL vary wildly in orientation and length even among neighboring OLs (Almeida et al., 2011; Chong et al., 2012; Tomassy et al., 2014). The unmyelinated space between internodes forms the node of Ranvier (see Figure 1B) (often referred to simply as “nodes,” hence the moniker internode, meaning “between nodes”). It is here that axonal signal propagation and current flow are maintained through membrane depolarization by high-density voltage-gated sodium channels, as the signal hops from node to node (this hoping forms the root of the term “saltatory” conduction, meaning to “jump” or “leap”) (Nave, 2010a). Had myelin not evolved, neurons would expend enormous amounts of energy maintaining sodium and potassium channel architecture and ion gradients along the axon’s entirety. Neuronal circuits would be rendered inefficient, unable to meet the demands of a higher order nervous system.

Beyond providing an insulating substance for proper salutatory conduction, myelin is also required for long-term axonal integrity and survival. Early work supporting the concept demonstrated long-term, progressive axonal degeneration following depletion of critical myelin component proteins in mice (Griffiths et al., 1998; Lappe-Siefke et al., 2003). Importantly, the mice did not display any overt dysmyelination phenotype, suggesting that myelin’s role as insulator and axonal support system were segregated (Griffiths et al., 1998; Lappe-Siefke et al., 2003; Saab et al., 2013). Further evidence came in 2012, when two independent laboratories published work supporting myelin’s ability to excrete and provide encapsulated axons with lactate – the metabolite being transported into the axon and there used to produce ATP (Fünfschilling et al., 2012; Lee et al., 2012b; Saab et al., 2013). In a similar vein, OLs were also shown to secrete exosomes in response to neuronal activity (Frühbeis et al., 2013). Exosomes were absorbed by the neuron’s axon (under the myelin sheath) and cell body, their protein, and/or RNA cargo internalized. The exosomes proved neuroprotective, supporting neuronal metabolism during times of stress (for example, nutrient starvation) (Frühbeis et al., 2013). Clearly, there exists an intimate relationship between the OL and axon that is essential to cellular integrity and survival, one that we have just now begun to explore.

### Myelin compaction

Myelin architecture, as classically captured through electron micrographs, is formed of concentric layers of compact membrane ensheathing an axon (Figure 1B). The innermost layer consists of an uncompacted inner tongue, and the outermost a similar uncompacted outer tongue (although, as we will see later, the inner tongue, and not the outer, is the engine driving radial myelination). The myelin’s lateral edge forms cytoplasm-heavy paranodal loops, which align and define the myelin-nodal border (see Figures 1B and 2 for detailed schematic of myelin structure) (Nave, 2010a). It is along these uncompacted regions that metabolites, such as lactate, are thought to be transported and then fed to the myelin encased axon (Saab et al., 2013).

Myelin compaction is driven in large part by myelin basic protein (MBP). Often compared to a spring, the protein binds opposing inner membranes drawing the two faces together. MBP then clusters into a dense fibrillary network. Likened to nuclear pores, the clustering dams access, allowing only select molecules to pass through. Thus, local protein mobility and concentrations are drastically reduced (Aggarwal et al., 2011, 2013; Bakhti et al., 2014). As a result, MBP deficient mice, an extremely well-studied dysmyelination model, do not produce compact myelin (Roach et al., 1983; Schain et al., 2014).

Unsurprisingly, compact myelin is relatively devoid of cytoskeleton (Aggarwal et al., 2011; Snaidero et al., 2014). In the absence of actin and tubulin, engines of morphological change, the sheath appears in the desired static state. Although, as we shall see, this is somewhat of an illusion – the engine can be rekindled and myelin profiles can change in response to a shifting environment (Liu et al., 2012; Gibson et al., 2014; Snaidero et al., 2014).

### The cytoskeleton in oligodendrocytes and myelin membrane

Such radical morphological change in the OL, from bipolar to myelin-producing cell, demands a dynamic cytoskeleton. The OL houses two major cytoskeletal components: microtubules and microfilaments (the latter will hereafter be referred to as F-actin) (Bauer et al., 2009). Independently or in tandem, they form adaptive structures and give rise to an underlying architecture prepared for rapid and sustained growth. Both components are expressed to varying degrees and within distinct regions at all stages of differentiation *in vitro* (due to their ubiquitous nature, direct assessment *in vivo* is rare). In the coming section, we will explore their role in process outgrowth and myelin membrane formation, as well as describe numerous regulatory proteins governing cytoskeletal function.

### The cytoskeleton: F-actin and microtubules

**Figure 3:**
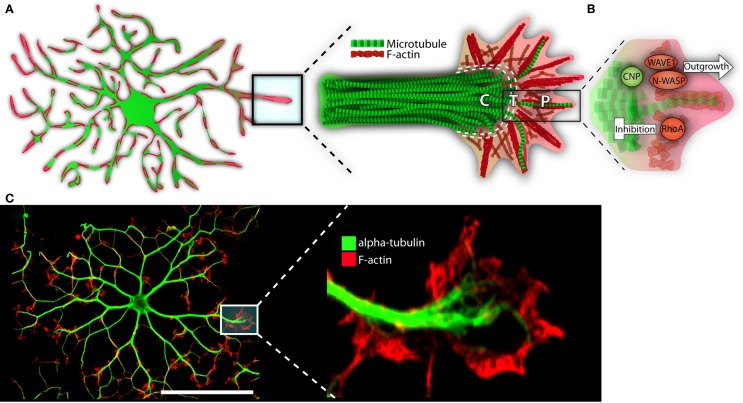
**The growth-like cone and cytoskeleton in maturing oligodendrocytes**. **(A)** Microtubules (depicted in green) run the length of OL processes in long parallel lines. F-actin (in red) is found throughout the OL, but is most heavily concentrated at the growing ends. These growing ends, or tips, are highly reminiscent of neuronal growth cones in both structure and cytoskeletal distribution. Neural cones have three distinct domains: a microtubule rich central (C) domain, an F-actin rich peripheral (P) domain, and a transient (T) domain where microtubules and F-actin overlap. We have overlaid these domains with the OL growth-cone-like structure to demonstrate similarities. In the schematic, microtubules (in green) invade the OL growth-cone centrally. Most do not advance much past this central point and are clearly delineated from the peripheral F-actin. The OL’s leading edge is replete with a meshwork of F-actin fibers (depicted in red). As well, parallel F-actin bundles (depicted as bundled long red strands), push against the membrane edge generating filopodia. A very few microtubules will penetrate the peripheral domain. In a neuronal cone, these exploratory microtubules are important for directed growth. **(B)** A basic schematic depicting various cytoskeletal assembly/remodeling proteins that drive or stall the oligodendrocyte’s growth cone. WAVE1 and N-WASP widen the cones lamellopodia, leading to process extension through F-actin branching and polymerization. CNP directs F-actin rearrangement, microtubule growth/bundling, as well as acts as a bridge between these two cytoskeletal components. CNP’s expression is strongly linked to OL morphogenesis; its ability to synchronize dynamic F-actin reorganization with microtubule polymerization and, therefore, process stabilization, is in large part responsible for OL outgrowth. RhoA, by contrast, acts as a stop-gate, preventing growth through generation of actomyosin contractile forces. Only when the RhoA pathway is deactivated can morphological development proceed. **(C)** Immunofluorescent representation of cytoskeleton and growth-like cone in the OL. The left panel depicts an immature OL with F-actin in red and α-tubulin in green. The right depicts a single growth-cone-like structure on the tip of an elongated OL process. P = peripheral, C = central, T = transient. Scale bar: 50 μm.

In the immature OL, F-actin is highly concentrated at the process’s leading edge. The edge is similar to that of a neuronal growth cone; it is formed of lamellipodia and filopodia, the latter extruding the surface (see Figure 3) (Rumsby et al., 2003; Fox et al., 2006). Splayed microtubules sit behind this highly active front. They invade from the more stable primary processes, themselves replete with bundled microtubules (Lunn et al., 1997; Song et al., 2001; Bauer et al., 2009). As the OL matures, and morphological complexity increases, microtubules display increasingly high levels of acetylated α-tubulin, indicating long-term stability for both microtubule and the process it inhabits (Lunn et al., 1997; Song et al., 2001; Lee et al., 2005). Taken together, cytoskeletal-mediated growth can be envisioned as follows: an F-actin rich OL “growth cone” leading the way, laying down a “track” for microtubules and the process to follow. As the cell matures, tubulin is acetylated, microtubules are stabilized, and the dense branched network is maintained (Song et al., 2001; Bauer et al., 2009).

As discussed, a shift from process outgrowth to membrane production results in a progressively sparse cytoskeleton. Following membrane compaction (*in vitro*), F-actin is relegated to the cell’s periphery, a region containing uncompacted membrane (a potential correlative for cytoplasm-heavy structures *in vivo*, such as the inner tongue) (Dyer and Benjamins, 1989a; Bauer et al., 2009; Aggarwal et al., 2011; Snaidero et al., 2014). Microtubules are longer lasting, forming a dense pattern of cytoplasm-rich tendrils that snake between larger pockets of compact membrane (Dyer and Benjamins, 1989a,b; Boggs and Wang, 2001). However, as the membrane continues to mature, microtubules are removed and the final product, a fully compact myelin sheath, is relatively devoid of both the cytoskeletal components (Dyer and Benjamins, 1989a,b; Boggs and Wang, 2001, 2004; Bauer et al., 2009; Aggarwal et al., 2011).

### The cytoskeleton: F-actin and microtubule regulating proteins

The OL’s leading edge is home to numerous F-actin assembly and remodeling proteins, such as the Arp2/3 complex, N-WASP, WAVE1, myosin II, and the small Rho GTPases, Rac1, Cdc42, and RhoA (Song et al., 2001; Fox et al., 2006; Kim et al., 2006; Bacon et al., 2007; Wang et al., 2012). Piece by piece, researchers have identified often unique and opposing roles for each.

The WASP family members, N-WASP and WAVE1, through interactions with Arp2/3, generate branched F-actin networks at the cell’s leading edge (Ridley, 2011). WAVE1-deficient OLs form fewer processes, and those formed lack discernable tip lamellipodia (Kim et al., 2006). *In vivo*, WAVE1 loss translates to fewer myelinated axons, while myelin thickness remains unaffected (Kim et al., 2006). Similarly, chemical inhibition of N-WASP leads to defects in OPC process extension and filopodia retraction. The cells also fail to initiate axonal ensheathment in chemically treated optic nerves (Bacon et al., 2007). Surprisingly, loss of either Rac1 or Cdc42 does not manifest any overt morphological defects in culture. Instead, OL-specific conditional knockouts display abnormal cytoplasmic accumulation in the myelin membrane’s inner tongue (Thurnherr et al., 2006). These seemingly anomalous findings fit with recent work describing myelin’s F-actin-rich inner tongue (one of the only structures sporting this cytoskeletal component in actively developing myelin *in vivo*) (Snaidero et al., 2014). During active myelination, the tongue is swollen and, as the myelin and animal matures, the tongue shrinks and F-actin is lost (Snaidero et al., 2014). Control of tongue size is dependent on phosphatidylinositol 3,4,5-triphosphate (PIP3) levels, a pathway that governs spatial activation of Rho GTPases (Hanna and El-Sibai, 2013; Snaidero et al., 2014). Regulation of Rac1 and/or Cdc42 levels or localization could therefore provide a mechanism for controlling tongue morphology through F-actin reorganization. It remains to be seen whether they are directly involved, although the loss of either Rac1 and/or Cdc42, leading to tongue swelling, strongly suggests it.

In stark contrast, RhoA inhibits OL morphogenesis through its downstream effector Rho-associated kinase (ROCK) (Wolf et al., 2001; Liang et al., 2004; Kippert et al., 2009; Rajasekharan et al., 2010; Wang et al., 2012). RhoA/ROCK’s role as morphogenic inhibitor is largely attributable to its activation of myosin II, which, in turn, leads to actomyosin-based hyper-contractility (Wang et al., 2008, 2012; Kippert et al., 2009). The RhoA/ROCK/myosin II pathway is thought to act as a gatekeeper for myelinating events – differentiation is triggered through downregulation of RhoA/ROCK’s activity, which loosens contractile forces and allows the OL to extend processes and eventually form membrane (Bauer and Ffrench-Constant, 2009; Rajasekharan et al., 2010; Wang et al., 2012).

Microtubule associating and/or binding proteins are also present in the OL (Bauer et al., 2009). One of the more intriguing is 2′,3′-cyclic nucleotide 3′-phosphodiesterase (CNP), a canonical myelin protein required for process outgrowth (Lee et al., 2005). Often used as a general marker for immature OLs, CNP binds tubulin heterodimers and drives microtubule assembly (Lee et al., 2005). Strikingly, when overexpressed in non-glial cells, radical morphological change ensues; the cells extend highly branched OL-like processes, microtubules bundle along the process length, and the F-actin network undergoes complete reorganization. In the case of actin, stress fibers are lost and growing processes, tipped with F-actin rich lamellipodia and filopodia, are formed (Lee et al., 2005).

Fortunately, high CNP levels are normally restricted to myelinating cells, as it appears necessary for a relentless drive toward growth (Lee et al., 2005; Bauer et al., 2009). In nascent myelin, CNP is localized to “loose” uncompacted wraps (Yin et al., 1997; Snaidero et al., 2014). Overexpression leads to exuberant process outgrowth and myelin production, followed by myelin compaction failure (Gravel et al., 1996; Yin et al., 1997). Recently, researchers have proposed a necessary equilibrium between CNP’s drive for growth and MBP’s desire to compact and stabilize the membrane (Snaidero et al., 2014). By playing one against the other, the OL ensures copious generation of “active” and “loose” membrane followed closely by membrane zippering, a final act in establishing compact myelin.

### Mechanisms of myelination

The past decade has seen advances in imaging technology and transgenic modeling that have allowed remarkable insight into the mechanical underpinnings of the OL. Detailed below are some of the more interesting findings relating to myelin sheath genesis, OL adaptability, and sculpting of the myelin landscape.

### All wrapped up: Mechanics of radial ensheathment and adaptive change

While researchers were long aware of the final myelin product – concentric layers of compact membrane – the mechanics involved in myelin wrapping were often best guesses or suppositions. Questions, such as the identity of the sheath’s leading edge, or initial direction of growth – inward or outward, radial, or lateral – remained unanswered. Over the years, various models were proposed, chief among them the “carpet-crawler” (Bauer et al., 2009; Snaidero et al., 2014). Here, upon axonal contact, the OL process spreads laterally, stretching the length of the future internode. Then, like rolling a carpet, the membrane loops under itself with progressive radial layers deposited one atop the other. Newer models both supported and contradicted varying aspects of the “carpet-crawler” (Pedraza et al., 2009; Sobottka et al., 2011). Contention focused on whether myelin (1) wrapped first as a relatively thin structure, and then expanded laterally across the axon, (2) laterally first, as seen in the “carpet-crawler” model, followed by radial wrapping, or (3) a mixture of the two.

In a study published in *Cell*, Snaidero and colleagues reconciled the conflicting points of view and offered a comprehensive vision of wrapping mechanics (Snaidero et al., 2014). The breakthrough rested largely with imaging technology and improved experimental technique. The authors employed pressure-freezing technology to preserve exquisite resolution of fine myelin structure. Three-dimensional myelin sheaths were generated from serial electron micrographs, and sheath minutiae explored, the authors able to track the ensheathment process from beginning to end (see Figure 2). As they describe, OLs first form a triangular membrane at axonal point of contact. Membrane expansion and wrapping then proceed in a coordinated bi-directional motion: simultaneous radial (around the axon) and lateral (along the axon) membrane expansion. Driving radial ensheathment is an F-actin-rich inner tongue, originating at the initial point of contact. The tongue pushes under pre-existing membrane, and remains juxtaposed with the axolemma, wrapping the axon in progressive layers (Figure 2B). In tandem, all myelin being generated moves laterally. The lateral edge, similar to the inner tongue, remains uncompacted and in constant contact with the axon. As myelination proceeds, the lateral edge of each layer halts at the nodal border, leading to the sequential generation of paranodal loops one behind the other (Figure 2C). Membrane compaction then begins in the outermost layers, moving inexorably inwards as the system matures. The unprecedented resolution in the Snaidero study allowed identification of cytoplasmic channels running throughout compacted myelin. The channels were shown to traffic materials for sustained growth at the leading edge. The authors also demonstrated capacity within the system for inner tongue reactivation and cytoplasmic channel reappearance, leading to thickening of the pre-existing myelin. The effect is PIP3-dependent, a pathway whose members are capable of driving OL maturation, as well as inducing hypermyelination in the adult brain (Flores et al., 2008; Narayanan et al., 2009; Goebbels et al., 2010; Snaidero et al., 2014).

That mature OLs retain the capacity to drive ensheathment fits perfectly with the model of white matter as adaptive and plastic. Recent evidence suggests that reshaping of the myelin landscape occurs not only through *de novo* events or replacement of dying OLs (see section on OPCs) but also through plasticity in the myelin sheath itself. In mice, forced activation of motor neuronal circuitry by means of optogenetic stimulation leads to increased myelin thickness in the stimulated region (Gibson et al., 2014). A gain in associated motor outcome was also dependent on increased myelin thickness – it was lost when the OL’s ability to produce myelin membrane was inhibited (although, it must be noted, the authors were unable to decidedly prove whether the thicker myelin was not simply the result of *de novo* myelination of previously unmyelinated axons) (Gibson et al., 2014). In contrast, areas of the pre-frontal cortex are thinly myelinated when mice are kept in isolation during critical periods of development (Makinodan et al., 2012). A similar thinning of myelin is observed in isolated adult mice, with social re-introduction leading to upregulation of myelin gene expression (Liu et al., 2012).

In a clever set of experiments, researchers created transgenic mice incapable of generating new OLs from adult OPCs (McKenzie et al., 2014). When given a novel motor task, the mice were unable to attain the same level of proficiency as their wild-type counterparts. It became apparent that *de novo* myelination, the result of newly formed OLs, was required for mastery of a novel motor skill, perhaps through a boost to the efficiency of underlying neuronal circuits. It is clear that OLs and the myelin they produce carry the tools to respond to shifts in higher order systems. Alterations in myelin thickness and/or *de novo* internode formation could mold signal speed in response to social and motor adaptation, adding another layer of control to an already highly refined system.

### To myelinate or not: Deciding to form internodes

But what of the switches regulating OL development? For example, how does an OL choose to myelinate an axon? It is an interesting question, as OLs undergo almost all phases of differentiation when cultured in isolation, and importantly, in the absence of axons. In fact, OLs are more than willing to myelinate fixed “dead” axons or engineered nanofibers (Rosenberg et al., 2008; Lee et al., 2012a, 2013). Further, the “dead” axons are myelinated to the same extent as the living, suggesting that dynamic exchange between OL and axon is not necessary for myelination *per se* (Rosenberg et al., 2008). These data point to a system whereby OLs are relatively autonomous in their drive to undergo differentiation, and will produce myelin given a suitable scaffold. But this is not to say that myelination occurs without reason. First, size regulates temporal order of myelination, with larger axons preceding smaller, both *in vivo* and in nanofiber cultures (Almeida et al., 2011; Lee et al., 2012a). A single OL will also myelinate more small axons relative to large (Almeida et al., 2011). In deciding which axons to myelinate, the OL further displays incredible adaptability. When zebrafish are manipulated to produce a greater number of large axons, OLs respond in kind by producing a greater number of sheaths (Almeida et al., 2011). The effect is not due to increase in OL number. Rather, normal myelination is achieved through adaptive change in OL morphology. The same OL simply extends more processes, though it must be said that a massive increase in total axon number does seem to precipitate a concomitant increase in OL number (Burne et al., 1996). There is also clear competition for axonal space between neighboring OLs – by reducing OL density, the number of internodes generated by any given OL increases (Chong et al., 2012). The effect seems mediated by repulsive OL membrane-bound cues, which allow for coordinated non-overlapping internode formation. Loss of one such cue, Nogo-A, results in increased myelination potentiation with associated exuberant myelin internode formation *in vivo* (Chong et al., 2012). Importantly, even here, the final global myelination pattern in the adult is not different with or without the restrictive cue. Rather, it leads to premature myelin expansion with an associated decrease in total mature OL number. This effect is most likely due to a percentage of the population being rendered redundant as they are outcompeted by uninhibited neighboring OLs.

But just how plastic is internode formation? While evidence overwhelmingly suggests ongoing myelination throughout life, the question remains: can mature OLs continue to generate internodes as the CNS requires? Recent live imaging experiments suggest that this is not the case. Instead, dynamic internode formation (the time allocated an OL to lay its first and last sheath) is limited to a temporal window – approximately 5 h in zebrafish – after which time, barring a small number of myelin sheath retractions, internode number and OL morphology remain static (Czopka et al., 2013). Even when the ability of OLs to generate internodes is altered (increased and decreased) through manipulation of Fyn kinase – a key integrator of multiple axo-glial signaling pathways regulating OL myelination potential through RhoA/ROCK – the time constraint remains in effect (Umemori et al., 1994; Liang et al., 2004; Laursen et al., 2009; Rajasekharan et al., 2009; Wake et al., 2011; Czopka et al., 2013). Coupled with the data on shifting myelin sheath thickness in the adult brain (see previous section), a general model emerges: newly generated OLs (both in developing and adult organisms) can adapt to their environment and produce the requisite number of sheaths within a relatively narrow time frame. Subsequent adaptive requirements placed on the mature OL, say in response to social and motor behavioral change, can then be made through alterations to the myelin sheath itself or through a separate *de novo* myelinating event requiring a newly generated OL.

Recent evidence also points to heterogeneity in longitudinal internode patterning within the murine cortex (Tomassy et al., 2014). Pyramidal neurons in the deeper cortical layers (V/VI) offered a stereotyped myelin profile, characterized by long myelin internodes interspersed with short nodes of Ranvier. Standing in stark contrast, neurons in the upper cortical layers (II/III) were defined by sparse and highly irregular internodes (from very short to very long), with stretches of bare axon far outside the typical range for a node. As the study’s authors note, these unmyelinated regions stretched upwards of 50 μm along the axon, particularly striking when a typical node measures 1–2 μm (Tomassy et al., 2014). And while an overarching pattern could be assigned to each layer (dense and long in the deep layers, sparse and erratic in the upper layers), adjacent axons themselves carried unique internode profiles, lending each individual neuron a myelin “signature.”

Beyond spatial determination, an OL’s myelin profile is intrinsically linked to the developmental stage at which it is born. Relative to “early” OPCs (birthed during development), OLs derived from “late” adult OPCs produce a greater number of internodes, but each of a shorter length. As described by Young and colleagues, individual OLs in young mice produced “~21 internodes of length ~76 μm” while OLs in more mature animals produced “~77 internodes per cell of mean length ~22 μm” (Young et al., 2013). Strikingly, while the number and length of myelin sheaths differed, the sum of all myelin lengths produced per cell was nearly identical regardless of temporal birthing. Each group simply reached maximal myelin output in an inversed manner (Young et al., 2013). OLs birthed during early development do, however, produce thicker myelin (more layers) than their adult counterparts. Precisely why there is a temporal shift in the OPC’s internode capacity (length, number, and thickness) remains a mystery. The findings do, however, resonate with the remyelination process – the focus of OL disease research – as remyelinated axons are characterized by both shorter and thinner myelin internodes (Blakemore and Murray, 1981; Franklin and Ffrench-Constant, 2008). Researchers have therefore suggested that the observed decrease in myelination capacity following a demyelinating event is not necessarily injury-related, but rather, is characteristic of an aged OPC (Young et al., 2013). While not the focus of this review, remyelination is the subject of intense study, driven in large part by the therapeutic promise of white matter regeneration and a need to understand why the process can stall or outright fail during disease. The reader is encouraged to refer to any of the number of recently published reviews detailing the subject [see reviews by Fancy et al. (2011), Franklin and Gallo (2014), and Franklin and Goldman (2015)].

### Sculpting the myelin landscape

The OL is clearly an adaptive cell, able to respond to shifts in axonal number and size. Myelination is therefore a process of pruning and sculpting, a fine-tuning of the OL’s underlying need to myelinate, as seen through distinct myelin patterning and profiles in the brain (Tomassy et al., 2014). It therefore becomes a question of balance, playing factors that inhibit and cut from the growing OL against local cues that serve to boost speed or extent of myelination. Many such signals have been identified and studied, such as ligands and secreted molecules present on axons and/or the OL itself, as well as molecules in the OL’s environment (the extracellular matrix). Some are inhibitory, such as Nogo-A (which we have already discussed) (Chong et al., 2012), LINGO-1 (Mi et al., 2005), and PSA-NCAM (Charles et al., 2000). Loss of inhibitory cues results in premature and exuberant myelination (Charles et al., 2000; Mi et al., 2005).

There are also enhancers and drivers of myelination. While no single molecule has yet been identified as a master regulator, there is direct evidence for various axonal ligands or secreted substances, such as glutamate and/or Neuregulin-1 (Nrg1), in regulating OL differentiation and extent of myelination (Brinkmann et al., 2008; Wake et al., 2011; Makinodan et al., 2012). Glutamate fits nicely with a paradigm for circuit activation wherein stimulation and axon firing, resulting in neurotransmitter release, signals to the OL that the axon is ready to myelinate (Charles et al., 2000; Wake et al., 2011). Nrg1’s story in the CNS is slightly more convoluted. For Schwann cells (myelin-producing cells of the peripheral nervous system), Nrg1 is absolutely critical for myelination (Michailov et al., 2004; Taveggia et al., 2005). However, defying expectations, a similar narrative did not evolve in the CNS – Nrg1 pathway disruption had minimal impact on overall CNS myelination (Brinkmann et al., 2008). Rather, it was Nrg1’s overexpression that gave rise to a phenotype: an increase in total number of myelinated axons (which the authors attribute to increased internodal length) coupled with hypermyelination (more myelin wraps around an axon) (Brinkmann et al., 2008). Interestingly, the latter observation supports recent findings for mice kept in isolation (see previous section “All Wrapped Up: Mechanics of Radial Ensheathment and Adaptive Change”). When mice were isolated, myelin sheaths in the pre-frontal cortex thinned, a phenomenon replicated by loss of Nrg1’s receptor ErbB3 (Makinodan et al., 2012). Further, social isolation led to decreased Nrg1 expression (Makinodan et al., 2012).

In 2013, researchers linked glutamate and Nrg1 to a co-operative myelin enhancing program dependent on neuronal signaling (Lundgaard et al., 2013). In the absence of Nrg1, OLs employed a baseline program to drive myelination independent of neuronal activity (hence their ability to myelinate fixed axons, or differentiate in isolation). However, when presented with Nrg1, OLs switched to an activity-dependent program, accelerating OL differentiation and driving myelination beyond baseline. In this paradigm, myelination relied on OL *N*-methyl-d-aspartate (NMDA) receptor activation (NMDA receptors bind glutamate), an effect largely mediated by axonal firing and the release of glutamate (Lundgaard et al., 2013). However, once Nrg1 activated, the OL’s pre-programed “activity-independent” mechanism shut down – myelination was now almost entirely dependent upon NMDA receptor activation. If NMDA receptors were blocked following Nrg1 exposure, myelination was severely stunted, dropping far below baseline, while OLs never exposed to Nrg1 were unaffected. The findings helped resolve the long standing and perplexing issue surrounding Nrg1’s apparent minor role in OL-mediated myelination. Nrg1 knockouts would not have undergone a switch from activity-independent myelination to Nrg1-activity dependence if the proteins were ablated. It is an excellent example of the complexities inherent to the system, and how a pathway’s significance to the myelination process may differ dramatically if the OL exists in isolation or an activity-rich environment.

## Concluding Remarks

The past few years have seen monumental shifts in our understanding of OL biology, due in large part to advances in transgenic and imaging technology. Importantly, we have overturned preconceived notions of the OLs as “cells in stasis.” Rather, OLs are dynamic, adaptive, and plastic, fully capable of responding to a changing environment – from the birthing of new cells to alterations in pre-existing myelin. OLs also provide a distinct myelin profile not only to individual neurons but entire neuronal networks, imparting an additional layer from which to sculpt CNS behavior. In light of these findings – paradigm shifts in their own right – it appears almost paradoxical that we are still detailing some of the most basic properties of the OL (for example, the mechanics of myelin wrapping). It is indicative of the relatively large gaps in knowledge that remain. Thankfully, with improved technology and a renewed interest, the scientific community has begun to address many long held questions and will undoubtedly continue to do so well into the future.

## Conflict of Interest Statement

The authors declare that the research was conducted in the absence of any commercial or financial relationships that could be construed as a potential conflict of interest.
